# Isolation of autochthonous non-white rot fungi with potential for enzymatic upgrading of Venezuelan extra-heavy crude oil

**DOI:** 10.1080/10242420701379908

**Published:** 2007-10-11

**Authors:** Leopoldo Naranjo, Hector Urbina, Angela De Sisto, Vladimir Leon

**Affiliations:** Unidad de Biotecnología del Petróleo, Centro de Biotecnología, Fundación Instituto de Estudios Avanzados (IDEA), C/Hoyo de la Puerta-Baruta, Sartenejas, Caracas 1080, Venezuela

**Keywords:** Extra-heavy crude oil, fungi, bioconversion, upgrading, polycyclic aromatic hydrocarbons, lignin-degrading enzyme system

## Abstract

The increasing world demand for fuels makes it necessary to exploit the largest reserve of extra-heavy crude oil (EHCO) of the Orinoco Oil Belt from Venezuela. We propose the use of extracellular oxidative enzymes, in particular, lignin-degrading enzyme systems (LDS) of fungi, for enzymatic improvement of EHCO. Autochthonous non-white rot fungal strains able to use EHCO, and several polycyclic aromatic hydrocarbons (PAHs) as sole carbon source and energy, were isolated from EHCO-polluted soils and identified as belonging to the genera *Fusarium*, *Penicillium* , *Trichoderma* , *Aspergillus* , *Neosartorya*, *Pseudallescheria, Cladosporium*, *Pestalotiopsis* , *Phoma* and *Paecillomyces*. Phenotypic and biochemical assays revealed the ability of these filamentous fungi to synthesize extracellular oxidative enzymes, and suggested a relationship between the LDS and EHCO bioconversion. This work reports, for the first time, the use of *o*-phenylenediamine dihydrochloride (OPD) as substrate to measure extracellular ligninolytic peroxidases (ELP) in culture broths of filamentous fungi (*Fusarium solani* HP-1), and constitutes the first formal study of the fungal community associated with the EHCO of the Orinoco Oil Belt.

## Introduction

The complementary use of biotechnology in the petroleum industry provides new tools to improve heavy and extra-heavy crude oil (EHCO). Increasing world demand for fuels (estimated at 520 million barrels of petroleum for 2007) makes it necessary to exploit the extensive deposits of the Orinoco Oil Belt from Venezuela, the biggest reservoir of EHCO in the world, estimated at 1.5 trillion barrels (PDVSA 2002). Besides being particularly rich in heteroatoms (nitrogen, sulfur and oxygen), and metals such as nickel and vanadium, the EHCO of the Orinoco Oil Belt contains a high concentration of resins and asphaltenes, the two most polar fractions and with the highest molecular weight of EHCO. The latter are responsible for EHCO's high viscosity, which makes it difficult to extract, transport and refine by conventional methods (León 1998; for reviews, see León & Kumar 2005). This work focuses on the development of the enzymatic improvement of these Venezuelan non-conventional hydrocarbons using the powerful extracellular oxidative lignin-degrading enzyme system (LDS) of fungi as biological catalysts.

Lignin is a highly complex, stable and irregular polymer (Conesa et al. 2002), which is structurally similar to the resins and asphaltene molecules found in EHCO. The LDS, which has broad substrate specificity, includes a large range of oxidoreductases and hydroxylases, such as laccases (LACp) and high redox potential ligninolytic peroxidases (lignin peroxidase (LIPp), manganese peroxidase (MNPp), versatile peroxidase (VEPp) and others). These enzymes catalyze the oxidation of a highly diverse array of organic and inorganic compounds, such as polycyclic aromatic hydrocarbons (PAHs) and chlorinated aromatic compounds, DDT and other pesticides, dyes, cyanides, azides and cross-linked acrylic polymers (for reviews, see Leahy & Colwell 1990; Hammel et al. 1992; Vázquez-Duhalt et al. 1994; Bogan & Lamar 1996; Boonchan et al. 2000; Conesa et al. 2002; Van Hamme et al. 2003a; Foght 2004; Gianfreda & Rao 2004; Martín et al. 2004).

Enzymatic processes may provide an alternative to conventional methods for EHCO upgrading, having the advantage of being environmentally friendly and economically sound. LDS could change the physical properties of EHCO without the loss of carbon atoms, and keeping the calorific value of the hydrocarbons. Partial enzymatic oxidation of the internal aliphatic linkage (sulfides, esters, and ethers) and of PAHs, resins and asphaltene molecules can lead to a reduction in total aromaticity of EHCO, decreasing its viscosity and density levels (Kirkwood et al. 2004). Thus, the great interest in using fungal extracellular oxidative enzymes from LDS for enzymatic EHCO upgrading. If non-white rot fungal strains contain the LDS and it is involved in EHCO bioconversion, then EHCO addition as sole carbon source to culture broth should induce the extracellular oxidative activities from LDS. Filamentous fungi with the ability to produce extracellular oxidative enzymes on culture plates were identified by ABTS-oxidizing activity, as described previously by Saparrat et al. (2000), whereas the detection of extracellular ligninolytic peroxidase (ELP) activities in culture broths of filamentous fungi was performed using a rapid and efficient method, the *o*-phenylenediamine dihydrochloride (OPD) test. OPD is a very stable chromogen, water-soluble and a sensitive substrate used for diagnostic applications and immunochemical technique to measure peroxidase activity (Wolters et al. 1976; Bovaird et al. 1982). Interestingly, preliminary assays revealed that some autochthonous non-white rot fungi associated with EHCO are able to synthesize extracellular oxidative enzymes from LDS; this, in turn, suggests that these enzymes are implicated in EHCO bioconversions.

The principal aim of this study was to isolate, identify, and characterize autochthonous fungal strains with high potential to metabolize PAHs and EHCO. These filamentous fungal strains will be used as potential biocatalytic agents, to design novel green processes aimed at exploiting and rationally using natural resources, such as the Orinoco Oil Belt, and guarantee sustainable development of Venezuela.

## Materials and methods

### Isolation of autochthonous fungus and culture conditions

EHCO-polluted soils samples (ca. 5 g) from Sucre and Miranda states (Venezuela) were tested. Initially, the strains isolated were screened by selecting for their abilities to grow on Czapek (Cz) minimal medium (Peláez et al. 1995; Naranjo et al. 2005) supplemented with 2% (w/v) of Carabobo (formerly Cerro Negro) EHCO emulsion as sole carbon source (Cz^P^). Spores from fungal strains were grown on Cz^P^ for 7 days at 30°C.

### Phenotype assays from autochthonous fungal strains

Autochthonous fungal strains with the ability to grow on Cz^P^ were tested for their ability to grow on Cz supplemented with several PAHs (naphthalene, dibenzothiophene, phenanthrene, or pyrene) as sole carbon source and energy at a final concentration of 200 ppm. The plates were incubated for 7 days at 30°C in the dark, and growth of the fungal strains was analyzed. A scale based on colony growth was used for interpreting the results: non-growth (−); residual growth (−/−); weak growth (−); moderate growth (−); and good growth (−).

### Isolation of fungal genomic DNA

Spores from fungal strains were inoculated into MPPY medium (Montenegro et al. 1992; Naranjo et al. 2001, 2004) and incubated in an orbital shaker at 250 rpm for 36–48 h at 30°C. The resulting mycelium was recovered, filtered through Nytal filters, washed twice with 0.9% (w/v) NaCl, frozen with liquid nitrogen and stored at −80°C. Mycelium samples (ca. 500 mg) were treated with 1 mL of 0.18 M Tris/HCl pH 8.2; 10 mM EDTA; 1% SDS and 1 mL of phenol, and were incubated for 30 min at 50°C. Then, phenol–CIA treatment (phenol: chloroform:isoamyl alcohol, 25:24:1) was repeated until the interface was clear. Genomic DNA was precipitated with 2.5 vol of ethanol and 0.1 vol of 3 M sodium acetate (pH 3.2) and resuspended in Tris–EDTA (TE) buffer (Sambrook et al. 1989).

### Classic and molecular identification of autochthonous fungal strains

Fungal strains were classified by macro and microscopic studies of morphological characters (hyphae, conidia, chlamydospores, conidiogenus cells and conidiophores). The information was compiled in a taxonomic description for comparison with specialized literature. For molecular identification of autochthonous EHCO-degrading fungal strains, PCR amplifications of 28S rRNA gene were performed using the following specific primers: NL1 (forward): 5′-GCATATCAATAAGCGGAGGAAA AG-3′ and NL4 (reverse): 5′-GGTCCGTGTTTC AAGACGG-3′. PCR reactions were performed in an Applied Biosystems 2720 thermocycler using a final concentration of 0.25 μM of each deoxyribonucleotide triphosphate, and 100–300 ng of fungal genomic DNA as template. PCR conditions consisted of an initial denaturation at 95°C for 5 min, 40 cycles of amplification at 95°C for 35 s, annealing at 52°C for 30 s, extension at 72°C for 20 s, and final extension at 72°C for 10 min. The PCR product (600 bp) was doubly purified using the Wizard Genomic DNA Purification Kit (Promega, Madison, WI) and sequenced using BigDye™ Terminator v.3.1 kit (Applied Biosystems) on an ABI Prism™ 310 Genetic Analyzer (Applied Biosystems, Foster City, CA). Several DNA samples were sent to the UC Berkeley DNA Sequencing Facility (California, USA). All other nucleic acid manipulations were carried out by standard methods (Sambrook et al. 1989).

### Sequence analysis

*In silico* analysis of nucleotide sequences was performed using the Lasergene software package DNASTAR Programs (DNASTAR, Inc., UK), BLASTN (Altschul et al. 1997) and FASTA (Pearson & Lipman 1988).

### Detection of extracellular oxidative enzymes on culture plates

The ability of the fungal strains to produce extracellular oxidoreductases of LDS was performed by the 2,2′-azino-bis(3-ethylbenzothiazoline-6-sulphonic acid) (ABTS; Sigma-Aldrich) test as described previously by Saparrat et al. (2000). The chromogen ABTS is a very sensitive substrate that allows rapid screening of fungal strains producing the extracellular oxidative enzymes by means of a colorimetric assay at 420 nm (Saparrat et al. 2000). Each strain was processed in duplicate under controlled conditions at 30°C in darkness, for 8, 15 and 21 days. A scale based on the color intensity was used for interpreting the results: colorless (−) indicates no ABTS-oxidizing activity; low color intensity (+); moderate color intensity (++); and high color intensity (+++) indicating high ABTS-oxidizing activity.

### Detection of extracellular ligninolytic peroxidase activities in culture broths

Fresh spores were inoculated into MPPY medium and incubated in an orbital shaker at 250 rpm and 30°C. The mycelium grown-up was used as the inoculum for cultures in different conditions: (i) Cx medium (Cz medium with N-limited conditions); (ii) Cx supplemented with naphthalene as sole carbon source and energy; and (iii) Cx with 1% (w/v) of Carabobo-EHCO emulsion as sole carbon source and energy. Cultures were incubated at 30°C, and the supernatants collected at 48, 72, 96, 120, and 168 h post-inoculation and used for enzymatic assays. The detection of ELP activities was performed using the OPD oxidation test according to the manufacturer's instructions (Pierce; Rockford, USA) with some modifications. The reaction mixture contained 0.1 M citric/sodium phosphate buffer at pH 6.8; 0.75 mg mL^−1^ OPD; 20 μL of hydrogen peroxide 30% and 100 μL of sample (mycelium and EHCO-rest supernatant free) in a final volume of 1 mL. The mixture was incubated at room temperature for 30 min. OPD oxidation yields a soluble end product which is yellow–orange in color, and was monitored over 30 min at 490 nm using a Perkin-Elmer Instruments Lambda 35 UV/VIS spectrophotometer running UVwinlab version 2.85.04 2000 software. All reactions were carried out in duplicate, at least. The extinction coefficient used was 1.578 mM^−1^ cm^−1^, as determined previously by Romano et al. (2000). One OPD unit is defined as the amount of enzyme that oxidizes 0.01 μmol OPD per min (Romano et al. 2000). Protein concentration was determined by the Bradford assay (Pierce).

## Results and discussion

### Ability of isolated fungal strains to use EHCO and PAHs as sole carbon sources

**Figure 1:**
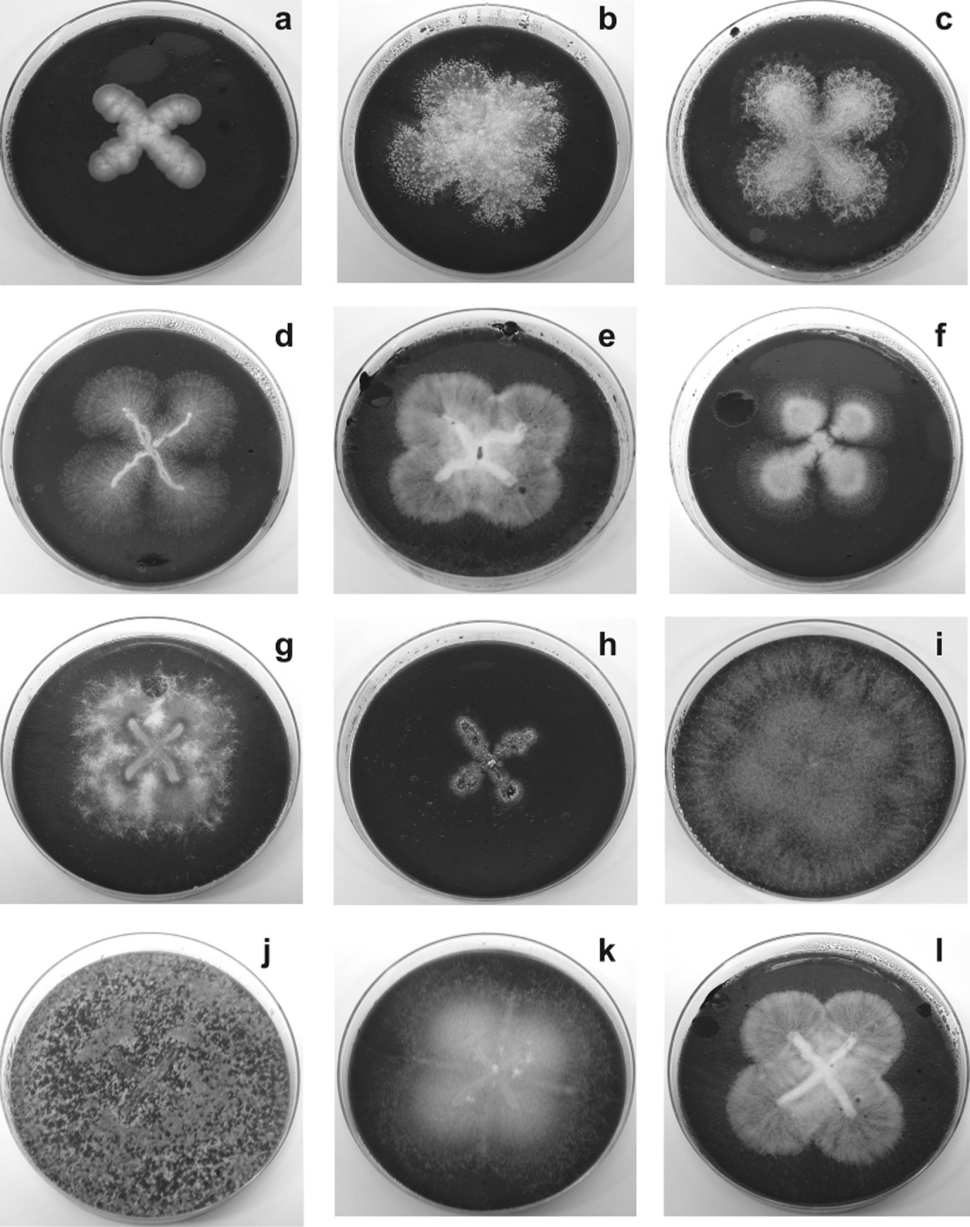
Several non-white rot fungal strains isolated from samples of EHCO-polluted soils and cultivated axenically. Note that these fungi were able to grow in Cz minimal medium supplemented with EHCO-emulsion as sole carbon source: (a) *Penicillium* sp. 4; (b) *Neosartorya spinosa* NS-2; (c) *Paecillomyces* sp.; (d) *Aspergillus terreus*; (e) *Pseudallescheria angusta* PA-2; (f) *Penicillium* sp. NRRL-28143 PN-2; (g) *Fusarium solani* HP-1; (h) *Pestalotiopsis palmarum*; (i) *Trichoderma viride* TV-1; (j) *Trichoderma inhamatum*; (k) *Fusarium proliferatum* FP-2; (l) *Pseudallescheria angusta* PA-1.

The potential of extracellular oxidative enzymes from LDS in remediation of polluted soils and oxidation of high-molecular-weight PAHs has been extensively described for white rot fungi (for a review, see Leahy & Colwell 1990; Conesa et al. 2002; Van Hamme et al. 2003a,b; Gianfreda & Rao 2004). However, very little is known about non-white rot fungi involved in PAH bioconversion. EHCO-polluted soils were used to isolate auto-chthonous fungal strains able to metabolize EHCO, yielding 45 different fungal strains, which were cultivated axenically and screened by selecting for the ability to grow on Cz^P^ (Figure 1). These strains were then tested for their ability to grow on Cz medium supplemented with several PAHs as sole carbon source and energy. This revealed that 20 different strains metabolized EHCO and various PAHs, indicating that it was possible to isolate filamentous fungi that grow under these extreme conditions using adequate selection techniques (Table I). These strains were retained for later identification. The results obtained through phenotype assays suggested that there is an enormous genetic diversity hidden in the crude oil-polluted soil linked to the PAHs and EHCO bioconversion. The enzymatic battery potentially available in this auto-chthonous genetic reservoir waits to be uncovered and used further in novel green processes.

**Table I:** Identification and phenotypic characterization of autochthonous non-white rot fungus isolated from EHCO-polluted soils.

						Czapek minimal medium supplemented with PAHs as sole carbon source						
						
No.^a^	Strain (classic and molecular identification)	Family	Order	No. CVCM^b^	Source	Control +	Control −	Pyrene	Phenan.	DBT	Naph.	Cyclohexane
1	*Fusarium solani* (Mart.) Sacc., HP-1	Hypocreaceae	Hypocreales	1784	Sucre	+++	+/−	++	++	+	++	−
2	*Fusarium proliferatum* (Matsush.) Nirenberg & Nirenberg, FP-1	Hypocreaceae	Hypocreales	1785	Sucre	+++	−	+	++	++	++	++
3	*Cladosporium sphaerospermum* Penz.	Mycosphaerellaceae	Dothidiales	1786	Sucre	++	−	+	+	+	+	*
4	*Pestalotiopsis palmarum* (Cooke) Steyaert.	Amphisphaeriaceae	Xylariaceae	1787	Sucre	+++	−	+	+	+	+	*
5	*Neosartorya spinosa* (Raper & Fennell) Kozak., NS-1	Trichocomaceae	Eurotiales	1788	Sucre	+++	−	++	+	+	++	+
6	*Neosartorya spinosa* (Raper & Fennell) Kozak., NS-2	Trichocomaceae	Eurotiales	1789	Sucre	+++	−	+	++	++	++	+
8	*Penicillium* sp. NRRL-28143, PN-1	Trichocomaceae	Eurotiales	1790	Sucre	+++	+/−	++	+/−	+/−	+	++
13	*Aspergillus fumigatus* Fresen., AF-1	Trichocomaceae	Eurotiales	1791	Miranda	+++	+/−	+	+	+	+	++
15	*Pseudallescheria angusta* (Malloch & Cain) McGinnis, A.A. Padhye & Ajello, PA-1	Microascaceae	Microascales	1792	Miranda	+++	+	+	+	+	+	++
23	*Penicillium* sp. NRRL-28143, PN-2	Trichocomaceae	Eurotiales	1793	Miranda	++	+/−	++	+	+	++	++
26	*Aspergillus fumigatus* Fresen, AF-2	Trichocomaceae	Eurotiales	1794	Miranda	+++	−	+	+	+	+	++
28	*Trichoderma viride* Pers., TV-1	Hypocreaceae	Hypocreales	1795	Miranda	+++	−	+	+	−	+	+
31	*Trichoderma viride* Pers., TV-2	Hypocreaceae	Hypocreales	1796	Miranda	+++	−	+	+	+	+/−	++
32	*Phoma glomerata* (Corda) Wollorw. & Hochapfel, Z.	Dothideomycotidae	Pleosporales	1797	Miranda	+++	−	+	−	+	++	++
35	*Trichoderma inhamatum* Veekamp & W. Gams	Hypocreaceae	Hypocreales	1798	Miranda	+++	−	++	++	++	++	+
36	*Aspergillus terreus* Thorn	Trichocomaceae	Eurotiales	1799	Miranda	+++	−	++	++	++	++	++
37	*Penicillium* sp.	Trichocomaceae	Eurotiales	1800	Miranda	++	−	++	++	++	++	++
38	*Paecillomyces* sp.	Trichocomaceae	Eurotiales	1801	Miranda	+++	+/−	++	++	++	++	++
39	*Pseudallescheria angusta* (Malloch & Cain) McGinnis, A.A. Padhye & Ajello, PA-2	Microascaceae	Microascales	1802	Miranda	+++	+/−	+	+	+	+	++
43	*Fusarium proliferatum* (Matsush.) Nirenberg & Nirenberg, FP-2	Hypocreaceae	Hypocreales	1803	Miranda	+++	−	+	++	+	−	++

a Accession number: Mycoplasm Bank of the Petroleum Biotechnology Unit of IDEA Foundation.

b Accession number: Venezuelan Centre of Microorganisms Collections (CVCM).

* The characterization was not done in cyclohexane.

Phenan: phenanthrene; DBT: dibenzothiophene; Naph: naphthalene. Residual growth (+/−); weak growth (+); moderate growth (++); good growth (+++).

### Identification of fungi isolated from crude oil-polluted soil

The 20 strains previously selected were identified through both classic and molecular techniques. Interestingly, the result showed that all strains isolated from EHCO-polluted soils were non-white rot fungi (Table I), and have been reported previously as ubiquitous soil-inhabiting saprophytes (Domsch et al. 1980). They belong to six predominant genera: *Fusarium* (3 spp.), *Penicillium* (3 spp.), *Trichoderma* (3 spp.), *Aspergillus* (3 spp.), *Pseudallescheria* (2 spp.), and *Neosartorya* sp. (2 spp.), teleomorph form of *Aspergillus fischeri* var. *spinosus*. Other strains isolated belonged to other non-predominant genera: *Cladosporium* (1 sp.)*, Paecillomyces* (1 sp.), *Pestalotiopsis* (1 sp.), and *Phoma* (1 sp.). Strains of the genera *Aspergillus*, *Cladosporium*, *Fusarium*, *Neosartorya*, *Paecillomyces*, *Penicillium*, *Phoma*, and *Pseudallescheri*, have previously been reported as culturable hydrocarbon-degrading fungi (Davis & Westlake 1979; April et al. 1998; Prenafeta-Boldú et al. 2001, 2002, 2004, 2006; Luykx et al. 2003; Plaza et al. 2003; Chaillan et al. 2004; Veignie et al. 2004; Verdin et al. 2005, 2006). However, this is the first report of the isolation of the filamentous fungus *Pestalotiopsis palmarum* from crude-polluted soil and its ability to metabolize PAHs and EHCO. The autochthonous fungi isolated (Table I) have been deposited in the culture collection of the Petroleum Biotechnology Unit of IDEA Foundation and the Venezuelan Centre of Microorganisms' Collections (Centro Venezolano de Colección de Microorganismos – CVCM).

### Evidence for the production of extracellular oxidative enzymes

Several extracellular oxidative fungal enzymes from LDS have been involved in the degradation of diverse recalcitrant and xenobiotics compounds (Vázquez-Duhalt et al. 1994; Saparrat et al. 2000; Alcalde et al. 2002, 2006; Gianfreda & Rao 2004). In order to detect the presence of extracellular oxidative enzymes in the isolated fungal strains, ABTS-oxidizing activity was analyzed by measuring the color intensity (oxidation of ABTS) of the agar medium. This revealed that extracellular oxidoreductases were present in 72% of the fungal strains tested (Table II). According to the scale described in Materials and methods, the filamentous fungi were grouped as: (i) species without extracellular ABTS-oxidizing activity: *Cladosporium sphaerospermum*, *Fusarium proliferatum* FP-1, *Neosartorya spinosa* NS-1 and NS-2 and *Trichoderma inhamatum* (similar levels were obtained with *Saccharomyces cerevisiae* used as negative control); (ii) species with low ABTS-oxidizing activity: *Penicillium* sp. NRRL-28143 PN-1 and PN-2 and *Penicillium* sp. 4, *Pseudallescheria angusta* PA-1 and PA-2, *Trichoderma viride* TV-1 and TV-2; (iii) species with moderate ABTS-oxidizing activity: *Aspergillus fumigatus* and *Aspergillus terreus*; and (iv) species with high ABTS-oxidizing activity: *Fusarium proliferatum* FP-2 and *Fusarium solani* HP-1, *Paecillomyces* sp. and *Pestalotiopsis palmarum*. This last group of non-white rot fungi showed similar levels of ABTS-oxidizing activity to that observed in the white rot fungus *Pleurotus ostreatus*, used as a positive control at 15–21 days (Table II). Previous studies have reported the genera *Fusarium* and *Paecillomyces* as hydrocarbon-degrading fungi (Plaza et al. 2003; Chaillan et al. 2004; Veignie et al. 2004; Verdin et al. 2005, 2006). *Fusarium*, *Paecillomyces* and *Pestalotiopsis* have also been reported as lignin-degrading fungi (Kapoor et al. 1978; Mishra et al. 1979; Norris 1980; Kamaya et al. 1981; Regalado et al. 1997; Kluczek-Turpeinen et al. 2003; Hao et al. 2006; Saparrat & Hammer 2006; Singh & Thakur 2006). Furthermore, before the establishment of the white rot fungus *Phanerochaete chrysosporium* as a model for study, *F. solani* was reported as the most vigorous microbial degrader of lignin among microorganisms isolated from soils and proposed as a useful model to study mechanisms of microbial transformations of lignocelluloses (Ohta et al. 1979; Norris 1980). More recent studies corroborated the lignin-degrading capabilities of *Fusarium* species (Regalado et al. 1997). Furthermore, several studies reported the lignin-degrading capabilities of *Paecillomyces fusisporus*, *P. varioti*, *P. inflatus*, and *Paecillomyces* sp. and the use of the latter for removal of color in pulp and paper mill effluents (Kapoor et al. 1978; Mishra et al. 1979; Kluczek-Turpeinen et al. 2003; Singh & Thakur 2006). Likewise, the ability of *Pestalotiopsis guepinii* to decolorize synthetic dyes and *Pestalotiopsis* sp. to produce laccase which efficiently decomposed lignocellulose have been described (Hao et al. 2006; Saparrat & Hammer 2006). Our results showed the high ligninolytic potential of the genera *Pestalotiopsis*, *Paecillomyces* and *Fusarium* (Table II). These experiments also represent an important step in selecting autochthonous fungal strains to be used in novel green processes development. Due to its high capacity to metabolize PAHs and EHCO and its high ligninolytic potential, strain HP-1 was selected for the following experiments.

**Table II:** Ligninolytic potential of autochthonous non-white rot fungus isolated from EHCO-polluted soils through ABTS test.

	Ligninolytic potential of non-white rot fungi determined by the ABTS test		
	
Strain	8 days	15 days	21 days
*Fusarium solani*, HP-1	+	+++	+++
*Fusarium proliferatum*, FP-1	−	−	−
*Cladosporium sphaerospermum*	−	−	−
*Pestalotiopsis palmarum*	−	++	+++
*Neosartorya spinosa*, NS-1	−	−	−
*Neosartorya spinosa*, NS-2	−	−	−
*Penicillium* sp. NRRL-28143, PN-1	+	+	+
*Aspergillus fumigatus*, AF-1	+	+	++
*Pseudallescheria angusta*, PA-1	−	+	+
*Penicillium* sp. NRRL-28143, PN-2	+	+	+
*Trichoderma viride*, TV-1	+	+	+
*Trichoderma viride*, TV-2	+	+	+
*Trichoderma inhamatum*	−	−	−
*Aspergillus terreus*	+	++	++
*Penicillium* sp.	+	+	+
*Paecillomyces* sp.	−	++	+++
*Pseudallescheria angusta*, PA-2	−	+	+
*Fusarium proliferatum*, FP-2	−	++	+++
*Pleurotus ostreatus* (+)	+++	+++	+++
*Saccharomyces cerevisiae* (−)	−	−	−

The presence of extracellular oxidoreductases was observed at 8, 15, and 21 days. Colorless (−); low color intensity (+); moderate color intensity (++); and high color intensity (+++).

### The relationship between LDS and EHCO bioconversion

**Figure 2:**
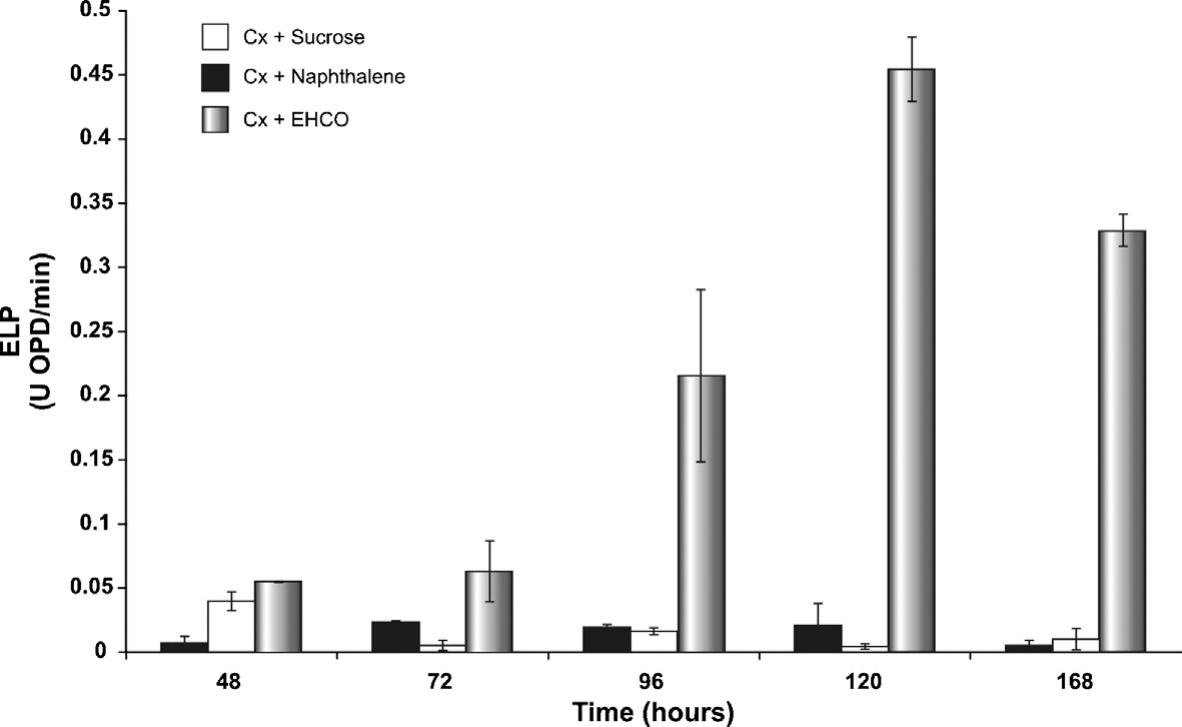
Detection of extracellular ligninolytic peroxidases (ELP) activities in culture broth of *Fusarium solani* HP-1 by the OPD test.

Lignin is a structurally similar polymer to the resins and asphaltenes, molecules responsible for the high viscosity and density of EHCO. For this reason, the relationship between LDS and EHCO bioconversion is of interest. The first step in lignin biodegradation is oxidation by hydrogen peroxide catalyzed by ELP. Among ELP, LIPp is able to directly oxidize non-phenolic units, MNPp and LACp oxidize preferentially phenolic units, but also act on nonphenolic units when mediators, such as ABTS, are present in the reaction mixture, whereas VEPp is able to combine the catalytic properties of LIPp and MNPp (Martínez et al. 1996; Ruiz-Dueñas et al. 1999; Saparrat et al. 2002; Gianfreda & Rao 2004; Martínez et al. 2005). If the LDS of strain HP-1 is involved in EHCO bioconversion, then EHCO addition as sole carbon source to culture broth might induce the ELP activities. In order to test this hypothesis, ELP activities in the culture broth of strain HP-1 were measured using OPD as the substrate. HP-1 strain was grown-up as described in Materials and methods. Surprisingly, the results showed that the ELP activities were present at the same basal levels when either sucrose (control) or naphthalene was used as sole carbon and energy source (Figure 2). However, the ELP activities were strongly induced when EHCO was used as sole carbon and energy source. Under these conditions, the ELP activities increased from 48 to 120 h, being nearly 3-, 12- and 20-fold higher at 72, 96, and 120 h, respectively, compared with a control (Figure 2). A decrease of ELP activities from 120 to 168 h was subsequently observed, but always showing significantly higher levels than the control. This substantial increase of ELP activities is probably due to the polymeric nature of EHCO. Unlike naphthalene, that represents a family of molecules constituted of just two benzene rings, EHCO is comprised of a diverse collection of aromatics and PAHs, some substituted with saturated hydrocarbons and heteroatoms, which are typical components of resins and asphaltenes, the most polar and highest molecular-weight fractions found in EHCO (León & Kumar 2005). Apparently, the bioconversion of compounds, such as lignin and petroleum, requires synergy of a large diversity of ELP with broad substrate specificity. Therefore, induction of ELP activities will be directly proportional to the complexity of the substrate. These results strongly suggest that: (i) HP-1 strain is able to synthesize ELP; (ii) ELP activities are strongly induced by EHCO and could be involved in EHCO bioconversion; and (iii) a putative relationship between LDS and EHCO bioconversion exists. Interestingly, preliminary studies aimed at improving EHCO with fungal biocatalytic agents, showed a decrease in the 500°C+ residue fraction with a significant increase in the distillate fraction (data not shown). The LDS could also be used in environmental biocatalysis for: (i) enzymatic bioremediation of xenobiotics, recalcitrant compounds, and petroleum-polluted soils; (ii) enzymatic recovery of sludge/oil spills; and (iii) renewable and clean energy production (Alcalde et al. 2006). Our next goal is the identification of the key genes and enzymes of the ELP family involved in the enzymatic bioconversion and improvement of Orinoco Oil Belt EHCO.
